# IgG4-related inflammatory pseudotumor of the renal pelvis involving renal parenchyma, mimicking malignancy

**DOI:** 10.1186/s13000-016-0460-z

**Published:** 2016-01-22

**Authors:** Ho Gyun Park, Kyoung Min Kim

**Affiliations:** Department of Urology, Chonbuk National University Medical School, Jeonju, Republic of Korea; Department of Pathology, Chonbuk National University Medical School, Research Institute of Clinical Medicine and Research Institute for Endocrine Sciences, Jeonju, Republic of Korea

**Keywords:** IgG4-related disease, IgG4, renal pelvis

## Abstract

**Background:**

IgG4-related disease is a recently recognized systemic disease characterized by storiform fibrosis with infiltration of IgG4-positive plasma cells. In rare incidences, IgG4-related renal disease can present as a solitary mass lesion at renal pelvis and can pose a diagnostic challenge since these lesions mimic malignancy. Herein, we present a rare case of IgG4-related disease presenting as inflammatory pseudotumor lesion, involving the renal pelvis and also neighboring renal parenchyma.

**Case presentation:**

A 75-year-old man with no history of IgG4-related disease underwent computed tomography (CT) scan for evaluation of prostatic cancer. The CT scan incidentally revealed a mass lesion located at the right renal pelvis. Radiologic findings were highly suggestive of malignancy. Therefore, the patient underwent right nephroureterectomy. Microscopically, the mass lesion showed storiform fibrosis with diffuse and intense inflammatory cell infiltration. Infiltrating cells were mainly histiocytes and plasma cells. Tubulointerstitium adjacent to the lesion also showed fibrosis with abundant plasmacytic infiltration. Immunohistochemical staining revealed the presence of IgG4-positive plasma cells in both the mass lesion and tubulointerstitium (mean of 94/HPF per field).

**Conclusion:**

Considering these findings, we diagnosed the mass lesion as IgG4-related inflammatory pseudotumor of the renal pelvis. In patients with renal pelvic masses, IgG4-related inflammatory pesudotumor should be considered in the differential diagnosis to avoid unnecessary surgical intervention.

## Background

IgG4-related disease is a recently recognized systemic disease characterized by advanced scleral fibrosis with extensive infiltration of IgG4-positive plasma cells [1]. IgG4-related disease has been reported to affect various organs, such as pancreas, salivary gland, lung, bile duct, breast, and prostate [2]. Among these sites, the pancreas is the most common organ of involvement. IgG4-related disease affecting the renal pelvis is usually characterized by the thickening of the pelvic wall [3]. However, in rare incidences, the disease can present as a solitary mass lesion and can often pose a diagnostic challenge since these lesions mimic malignancy, especially urothelial carcinoma of the renal pelvis [4, 5]. Herein, we present a rare case of IgG4-related disease presenting as an inflammatory pseudotumor involving renal parenchyma and pelvis in a patient with no history of IgG4-related systemic disease. Histologically the lesion featured storiform fibrosis, diffuse lymphoplasmacytic inflammatory cell infiltration, and obliterative phlebitis.

## Case presentation

**Fig. 1:**
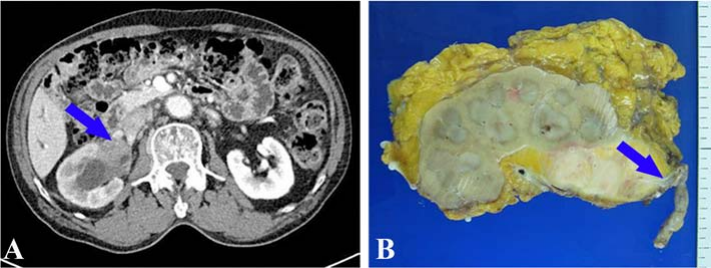
(**a**) CT scan show 5-cm-wide mass lesion located at the right renal pelvis (arrow). (**b**) Gross examination of the resected specimen showing relatively well circumscribed whitish firm mass. The mass lesion is mainly located at the renal pelvis and blocking the ureter pathway as the ureter is seen in the right side of the mass lesion (arrow). Also renal parenchyma adjacent to the mass lesion seems to be involved

A 75-year-old man, with no history of IgG4-related disease or any other autoimmune disease such as, systemic lupus erythematosus or Sjiogrens syndrome, was diagnosed with prostatic adenocarcinoma by needle biopsy. For further evaluation, computed tomography (CT) scan was performed. The CT scan incidentally revealed a 5-cm-wide mass lesion located at the right renal pelvis. The lesion was in the renal pelvis, and as a result, hydronephrosis was also present (Fig 1a). Magnetic resonance imaging (MRI) was performed for further evaluation of the renal pelvic mass. Compared to the surrounding tissue, the mass showed isointensity on T1-weighted images and hypointensity on T2-weighted images. On both T1- and T2-weighed images, the mass showed relatively homogenous enhancement. The patient did not exhibit any abnormal urologic symptoms and laboratory data were within the normal range. Urine cytology was also evaluated but did not reveal any malignant cells. We technically could not undergo a percutaneous needle biopsy because the tumor was near the renal major vessels. Biopsy via ureteroscopic or laparoscopic access also could not be done either. Because as we were suspecting the mass was a malignant tumor and feared the biopsy itself may cause cancer seeding or urine extravasation. Thereafter, the patient underwent right nephroureterectomy without preoperative biopsy.

**Fig. 2:**
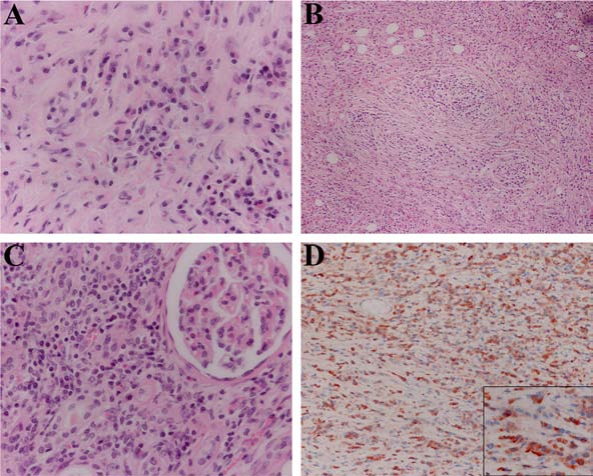
Histologic features of the mass lesion. (**a**) High power view showing storiform fibrosis with diffuse and intense inflammatory cell infiltration, mainly plasma cells and histiocytes. (**b**) Obliterative phlebitis is evident in some areas of the mass lesion. (**c**) Tubulointerstitium adjacent to the mass lesion showing fibrosis with abundant plasmacytic infiltration. (**d**) Immunohistochemically, IgG4-positive plasma cells infiltrated both the mass lesion and tubulointerstitium

Gross examination of the resected specimen showed a 5.2 cm sized whitish firm mass lesion. The mass lesion was mainly located at the renal pelvis blocking the ureter pathway and renal parenchyma adjacent to the mass lesion also seemed to be involved (Fig 1b). Microscopically, the lesion had no definitive margin and showed storiform fibrosis with diffuse and intense inflammatory cell infiltration. Infiltrating cells were mainly histiocytes and plasma cells. Granulomatous inflammation and obliterative phlebitis were also evident. Some areas showed lymphoid hyperplasia with germinal center formation. Tubulointerstitium adjacent to the lesion also showed fibrosis with abundant plasmacytic infiltration. Immunohistochemical staining revealed the presence of IgG4-positive plasma cells in both the mass lesion and tubulointerstitium (mean of 94/HPF per field) (Fig 2). Also we evaluated the ratio of IgG4-bearing plasma cells to IgG-bearing plasma cells and the ratio was approximately 58%. Considering these findings, we diagnosed the mass lesion as IgG4-related inflammatory pseudotumor of the kidney and renal pelvis. Serum IgG4 levels were normal (78.6 mg/dl) at 1 month after operation, and there was no evidence of IgG4-related disease at any other site during the 10 month follow up period, thereby obviating the need for any additional therapy.

## Conclusion

The first report indicating that serum IgG4 levels were elevated in patients with autoimmune pancreatitis and that pancreatic lesion of such patients showed infiltration of IgG4-positive plasma cells was published in 2001 [1]. Since then, reports have been published on the presence of IgG4-related lesion not only in the pancreas but also in other organs such as the bile duct, salivary glands, retroperitoneal tissue, and bone marrow. Thereafter, a new clinicopathologic entity called “IgG4-related systemic disease” was proposed [6].

In most cases, IgG4-related disease occurs in men (62 to 83%) and in those older than 50 years [7]. The disease can involve any organ, and the resultant symptoms are related to the affected site. Common sites involved by IgG4-related disease are the pancreas, hepatobiliary tract, salivary gland, orbit, and lymph node [7, 8]. The three key histopathologic findings of this disease are as follows: (1) dense lymphoplasmacytic infiltration, (2) storiform-type fibrosis, and (3) obliterative phlebitis [9]. If two of the three key histologic findings are present, the possibility of IgG4-related disease is strongly suggested pathologically. The most essential criterion to diagnose the disease is to the detection of IgG4-positive plasma cells in the lesion [7–9]. Although there have been variety of suggested cut-off points, ranging from >10 to >50 IgG4-positive plasma cells per high-power field (HPF) [9], most researchers are in agreement with >50 per HPF [10]. The diagnosis can be further assisted by the ratio of IgG4-positive plasma cells to IgG-positive plasma cells. A ratio >40% is considered to be highly suggestive of the diagnosis [10]. Although the presence of the characteristic histopathologic features and IgG4-positive plasma cells can provide strong supportive evidence for the diagnosis of IgG4-related disease, the definitive diagnosis should be made only by careful correlation with the results of clinical and imaging studies [9].

Kidneys are not spared in IgG4-related disease. Most of the reported cases of IgG4-related disease involving the kidney have synchronous or metachronous involvement in other organs [3]. However, in the present case, IgG4-related disease occurred in the kidneys in isolation. Clinically, some patients show non-specific symptoms such as fever, fatigue, anorexia, or abdominal pain, while others do not present any symptoms and are detected incidentally during laboratory tests or imaging studies [11]. Laboratory findings of patients with IgG4-related renal disease usually reveal hypergammaglobulinemia, hypocomplimentemia, or impaired renal function [12]. Serum IgG4 levels are elevated in about 80% of the patients [3]. According to previous reports, the suggested critical point of serum IgG4 level is 135 mg/dl [13]. An elevated serum IgG4 level may provide much assistance although it is not a necessity for the diagnosis [9]. Additionally, the disease activity and the number of involved organs could correlate with the serum IgG4 level [9]. Urinalysis of IgG4-related renal disease usually shows mild abnormalities. We did not consider the possibility of IgG4-related disease before the surgery, and therefore the preoperative serum IgG4 level was not evaluated. And all other laboratory findings including urine analysis were in normal limits.

Since most of the IgG4-related renal disease shows radiologic abnormalities, imaging study plays an essential role [3]. IgG4-related renal disease most commonly presents as multiple low-density nodules in one or both kidneys [8]. These kidney lesions usually show low intensity in contrast CT scans. And T2-weighted MRI scans of the lesions are typically hypointense [14].

In rare cases, IgG4-related renal disease can affect the renal pelvis and forms a single unilateral mass, as observed in our present case. Such lesions can pose a diagnostic challenge, because they mimic malignant tumors of the renal pelvis, especially if there is no other organ involvement. To the best of our knowledge, this is the third case of isolated renal pelvic IgG4-related disease to be reported in the English literature [4, 5]. The clinical and radiologic features of the previously reported cases are summarized in Table 1. Imaging studies in the two previously reported cases showed hydronephrosis and soft tissue mass lesion around the renal pelvic region, raising the suspicion of malignant tumor. Further, Wang et al. [4] reported that the mass showed high glucose metabolism in positron emission tomography/CT (PET/CT), making the diagnosis more difficult. In one of the reported cases, surgical intervention could be avoided because of the evaluation for patient’s serum IgG4 level. Unfortunately, the other patient’s serum IgG4 level was not evaluated and she underwent radical surgery.

**Table 1:** Clinical summary of reported cases

Author	Year	Sex	Age	Radiological features	Treatment
Yoshino et al. [7]	2012	71	M	CT- Hydronephrosis with soft tissue mass around the ureteropelvic junction	No surgical treatment Corticosteroid
Wang et al. [6]	2014	54	F	CT- Hydronephrosis with low-density renal pelvic mass	Nephroureteral cystectomy and retroperitoneal lymph node dissection
				MRI- Isointensity on T1-weigthed image and hypointensity on T2-weigthed image	Steroid therapy for 1 year after surgery
				PET/CT- High glucose metabolism	

In our present case, the imaging findings strongly suggested that the mass lesion was a malignant tumor. The radiologist mainly suspected the mass to be urothelial carcinoma of the renal pelvis. Moreover, the patient did not present any urologic abnormalities, such as hematuria or proteinuria. Since, IgG4-related disease was not included in the differential diagnosis, neither evaluation of serum IgG4 level nor preoperative biopsy was performed. Therefore, the patient underwent unnecessary radical excision.

A renal pelvic mass arising from IgG4-related disease should be differentiated from genitourinary malignancy, metastatic cancer, and lymphomas to avoid unnecessary surgery. However, unfortunately, this differentiation on the basis of radiologic criteria alone is extremely difficult. Several reports have shown that IgG4-related kidney disease has been mistaken for malignant mass and diagnosed only after invasive operations [15]. More than 90% of patients with IgG4-renal disease have concurrent involvement in other organs such as pancreas and salivary gland, while isolated kidney lesion is uncommon [16]. Evaluating other organs for masses or fibrotic lesions could provide an important clue to the diagnosis of the IgG4-related disease. For the same reason, we should pay more attention to the patients’ medical history and check for evidence of other organ involvement. If there is evidence of other organ involvement in the past or present, we should examine the serum IgG4 level and perform preoperative biopsy.

In conclusion, we have presented a rare case of IgG4-related inflammatory pseudotumor affecting both the renal parenchyma and pelvis. In our case, preoperative biopsy or checking of the serum IgG4 levels was not performed due to lack of evidence for IgG4-related disease. This case emphasizes the importance of considering that IgG4-related diseases can involve the kidney and give rise to mass-like lesions and mimic renal malignancy.

## Consent

Written informed consent was obtained from the patient for publication of this Case Report and any accompanying images. A copy of the written consent is available for review by the Editor-in-Chief of this journal.

## Abbreviations

CT: Computed tomography
MRI: Magnetic resonance imaging
HPF: High-power field
